# Observer- and sequence variability in personalized 4D flow MRI-based cardiovascular models

**DOI:** 10.1038/s41598-024-84390-4

**Published:** 2025-01-08

**Authors:** Belén Casas Garcia, Kajsa Tunedal, Federica Viola, Gunnar Cedersund, Carl-Johan Carlhäll, Matts Karlsson, Tino Ebbers

**Affiliations:** 1 Department of Health, Medicine and Caring Sciences, Linköping University, Linköping, Sweden; 2 Center for Medical Image Science and Visualization (CMIV), Linköping University, Linköping, Sweden; 3 Department of Biomedical Engineering, Linköping University, Linköping, Sweden; 4 School of Medical Sciences and Inflammatory Response and Infection Susceptibility Centre (iRiSC), Faculty of Medicine and Health, Örebro University, Örebro, Sweden; 5 Department of Clinical Physiology in Linköping, and Department of Health, Medicine and Caring Sciences, Linköping University, Linköping, Sweden; 6 Department of Management and Engineering, Linköping University, Linköping, Sweden

**Keywords:** Computer modelling, Cardiology, Mathematics and computing, Blood flow, Biomedical engineering

## Abstract

Subject-specific parameters in lumped hemodynamic models of the cardiovascular system can be estimated using data from experimental measurements, but the parameter estimation may be hampered by the variability in the input data. In this study, we investigate the influence of inter-sequence, intra-observer, and inter-observer variability in input parameters on estimation of subject-specific model parameters using a previously developed approach for model-based analysis of data from 4D Flow MRI acquisitions and cuff pressure measurements. The investigated parameters describe left ventricular time-varying elastance and aortic compliance. Parameter reproducibility with respect to variability in the MRI input measurements was assessed in a group of ten healthy subjects. The subject-specific parameters had coefficient of variations between 2.6 and 35% in the intra- and inter-observer analysis. In comparing parameters estimated using data from the two MRI sequences, the coefficients of variation ranged between 3.3 and 41%. The diastolic time constant of the left ventricle and the compliance of the ascending aorta were the parameters with the lowest and the highest variability, respectively. In conclusion, the modeling approach allows for estimating left ventricular elastance parameters and aortic compliance from non-invasive measurements with good to moderate reproducibility concerning intra-user, inter-user, and inter-sequence variability in healthy subjects.

## Introduction

Computational modeling of the cardiovascular system has gained increased relevance as a tool to understand physiological and pathophysiological changes, providing new insights into diagnosing and treating cardiovascular diseases. Patient-specific representations are being increasingly used, with model personalization being driven by individually acquired measurements that can be obtained invasively or non-invasively^1^. Among current cardiovascular modeling approaches, lumped parameter models have been proposed as a fast, relatively simple method to simulate global hemodynamics while keeping computational demands low^2^. Several lumped parameter modeling approaches have been developed for studying global hemodynamics. These generally combine lumped parameter models of the cardiovascular system with subject-specific input measurements derived from ultrasound measurements, cardiac magnetic resonance (CMR) acquisitions and invasive pressure catheter measurements in the heart or the aorta^3–6^.

Following personalization, the subject-specific model parameters can be used as cardiovascular biomarkers with potential application for diagnostic and prognostic purposes^7–13^. However, in order for these biomarkers to be reliable and successfully applied in healthcare, the methods for identifying them should be robust against errors and variability in these input data^14^. Previous studies have identified several sources of variability in computational models and highlighted the importance of considering errors in input measurements, also referred to as observational uncertainty, as a source of uncertainty in model-derived parameters^15–18^. Observational uncertainty is of interest in the evaluation of parameters derived from lumped parameter cardiovascular models, as personalization typically relies on a variety of anatomical and functional measurements that are susceptible to acquisition and analysis errors.

In a previous study, we presented an approach to personalize a lumped parameter model of the systemic circulation based on non-invasive measurements, which comprise CMR morphological images and flow data from four-dimensional magnetic resonance imaging (4D Flow MRI)^6^. The given approach allows for the identification of several cardiovascular parameters which are relevant for diagnostic purposes, such as parameters describing the contractile properties of the left ventricle in terms of its time-varying elastance^19^, and the compliance of the ascending aorta.

As a continuation of this study, we aimed to assess the reproducibility of these estimated parameters in relation to variability in the analysis and acquisition settings of the input images to the model. For this purpose, the parameters were investigated in a study of ten healthy volunteers examined with two 4D Flow MRI sequences with different scan parameters, which were then analyzed by two different observers in order to evaluate intra-observer, inter-observer and inter-sequence variability.

## Results

### Variability in input image-derived measures

The results of the intra-, inter-observer variability, and repeatability using the spoiled gradient echo (SGRE) and echo-planar imaging (EPI) 4D flow MRI sequences for the input measures to the model are shown in Table 1. None of the input parameters showed a significant mean difference in these investigations (*P* > 0.25 in all cases). The input parameter with the lowest coefficient of variation (CoV) in the intra-observer analysis was the effective orifice area of the aortic valve (EOA) (1.5%), while the lowest coefficient of variation in the inter-observer analysis corresponded to end systolic volume (ESV) (3.8%). In both the inter-observer analysis and when comparing the SGRE and EPI sequences, the LV outflow tract area (A_LVOT_) had the highest CoV (12 and 12%) among all the input measures, while ESV had the highest CoV in the intra-observer analysis (3%). For all input parameters, results of CoV from the inter-observer study and the comparison between the SGRE and EPI sequences were higher than their corresponding values from the intra-observer analysis. The variations in the length of the cardiac cycle between the acquisitions using the SGRE and EPI sequences had a CoV of 3.7% across all subjects, indicating a low variability in heart rate between the scans.

**Table 1:** Repeatability of input measures to the model derived from the MRI acquisitions comparing intra-, inter- observer analysis and the SGRE and EPI sequences.

Input image-derived measure	Intra-observer variability		
	Mean difference (− 1.96*SD, + 1.96*SD )	*P* value	CoV (%)
A_LVOT_ (cm^2^)	− 0.1 (− 0.63, 0.43)	0.91	2.8
ESV (mL)	0.0081 (− 6.1, 6.1)	0.91	3
EOA (cm^2^)	0.044 (− 0.11, 0.2)	0.73	1.5

	Inter-observer variability		
	Mean difference (− 1.96*SD, + 1.96*SD)	*P* value	CoV (%)
A_LVOT_ (cm^2^)	− 0.16 (− 2.7, 2.4)	0.97	12
ESV (mL)	0.81 (− 5.5, 7.2)	0.97	3.8
EOA (cm^2^)	− 0.25 (− 1, 0.51)	0.27	8.6

	SGRE-EPI		
	Mean difference (− 1.96*SD, + 1.96*SD)	*P* value	CoV (%)
A_LVOT_ (cm^2^)	− 0.1 (− 2.5, 2.3)	0.79	12
EOA (cm^2^)	0.16 (− 0.65, 0.97)	0.43	8.6
T (s)	− 0.0035 (− 0.12, 0.11)	0.73	3.7

For each input, the mean difference and the limits of agreement ($\overline{d} \pm 1.96SD)$, the *p*-value comparing the results from the two analyses/observers/sequences, and the coefficient of variation (CoV) are presented. A_LVOT_: left ventricular outflow tract area, ESV: left ventricular end systolic volume, EOA: effective orifice area of the aortic valve, T: length of cardiac cycle.

**Fig. 1:**
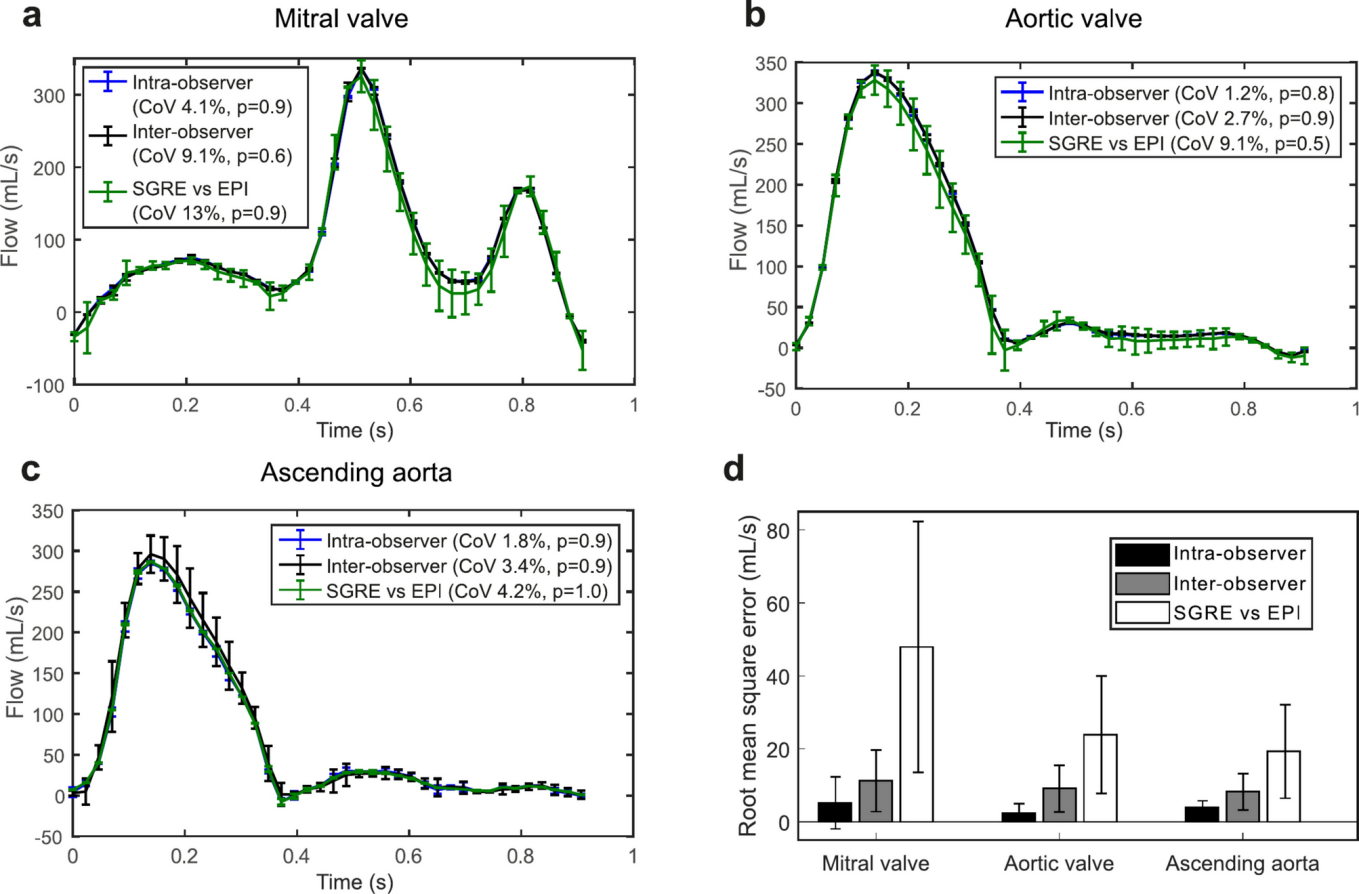
Variability in the volumetric flow waveforms used as input to the model in the mitral valve (**A**), aortic valve (**B**) and ascending aorta (**C**) of a representative subject. The curves and the error bars represent the mean and the standard deviation ($\pm$SD) of the volumetric flows for one subject and two different analyses corresponding to the intra-user variability study (blue), inter-user variability study (black) and SGRE-EPI comparison (green). The legend shows the coefficient of variations (CoV) and *p*-values of the net flow volumes, calculated from all 10 subjects. (**D**) shows the root mean square error (RMSE) of the volumetric flow waveforms across the ten study subjects at the selected measurement sites for these variability studies.

Figure 1 illustrates the variability in the volumetric flow waveforms used as input to the model for the intra- and inter-observer analysis and the SGRE-EPI sequence variability. Comparison of the root mean squared error (RMSE) for the input flow waveforms at the mitral valve, the aortic valve and the ascending aorta across all subjects revealed that, on average, the RMSE at the locations of interest was highest between the flow waveforms extracted from the SGRE and the EPI acquisition data, in comparison with the RMSE values found in the intra- and inter-observer analysis. Similarly, RMSE values in the input flow waveforms as a result of inter-observer variability were higher than those due to intra-user variability. The volumetric flows depicted in Fig. 1A–C exemplify the higher variability in the input flow waveforms resulting from the inter-observer analysis, as well as from comparison of SGRE and EPI sequences for one subject. In computing net flow volumes (Supplementary Tables 4), there were no significant differences for any of the sources of variation (*p* > 0.5), and the coefficients of variation for the total mitral flow were 4.1 and 9.1% for the intra- and inter-observer variability, respectively. For the aortic valve, these coefficients of variation were 1.2 and 2.7%, and for the ascending aorta 1.8 and 3.4%. Comparison of the net flow volumes calculated from the SGRE and EPI data yielded coefficients of variations of 13, 9.1 and 4.2% for the mitral valve, the aortic valve and the ascending aorta, respectively.

### Intra- and inter-observer variability in model parameters

**Fig. 2:**
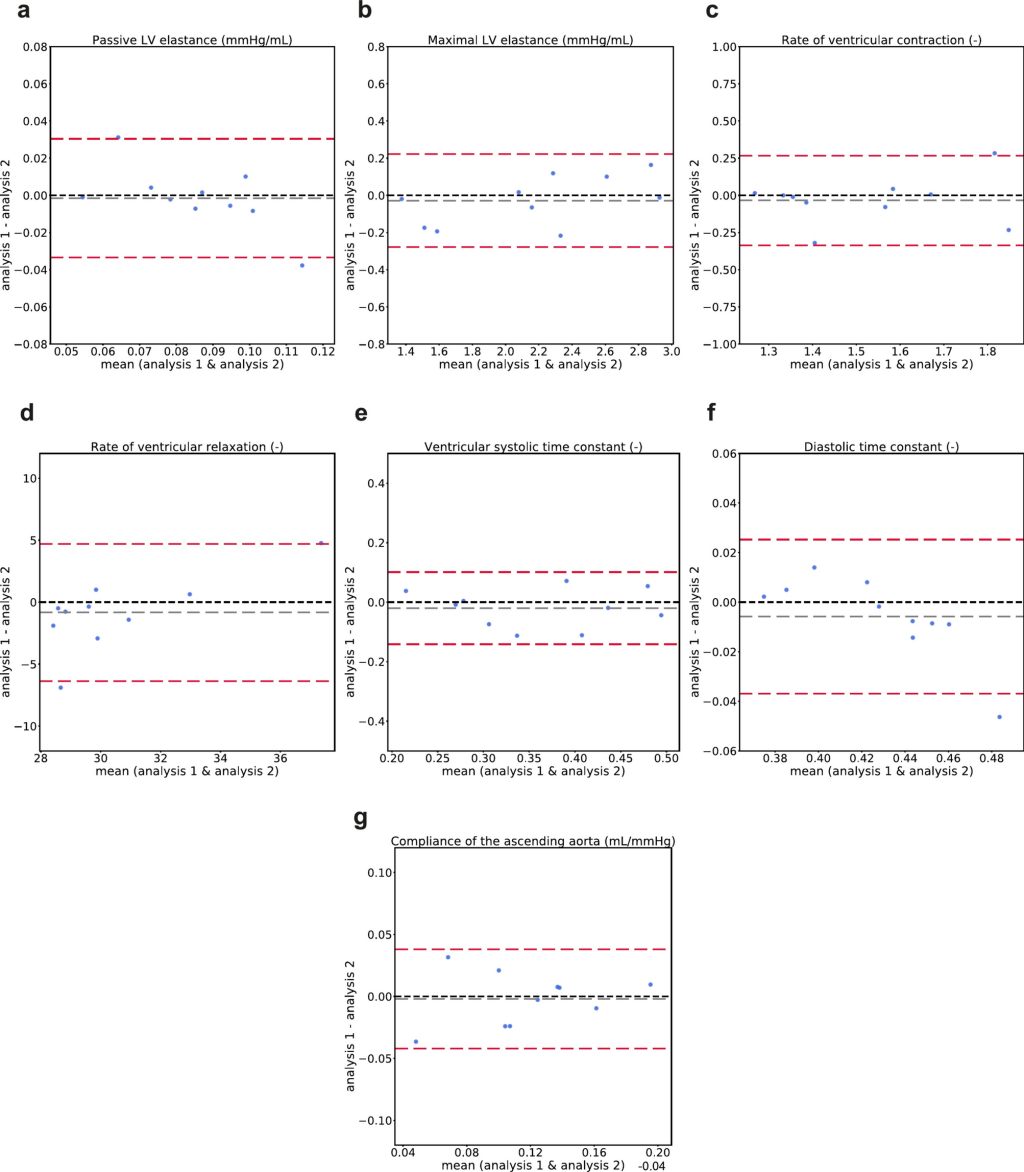
Bland–Altman plots showing agreement in the estimated model parameters using model inputs from the intra-observer variability study for **a**) passive left ventricular elastance, **b**) maximal left ventricular elastance, **c**) rate of ventricular contraction, **d**) rate of ventricular relaxation, **e**) ventricular systolic time constant, **f**) ventricular diastolic time constant, **g**) compliance of the ascending aorta. Grey dotted lines represent bias ($\overline{d}$) and red dotted lines represent limits of agreement ($\overline{d} \pm 1.96SD)$. LV: left ventricle.

**Fig. 3:**
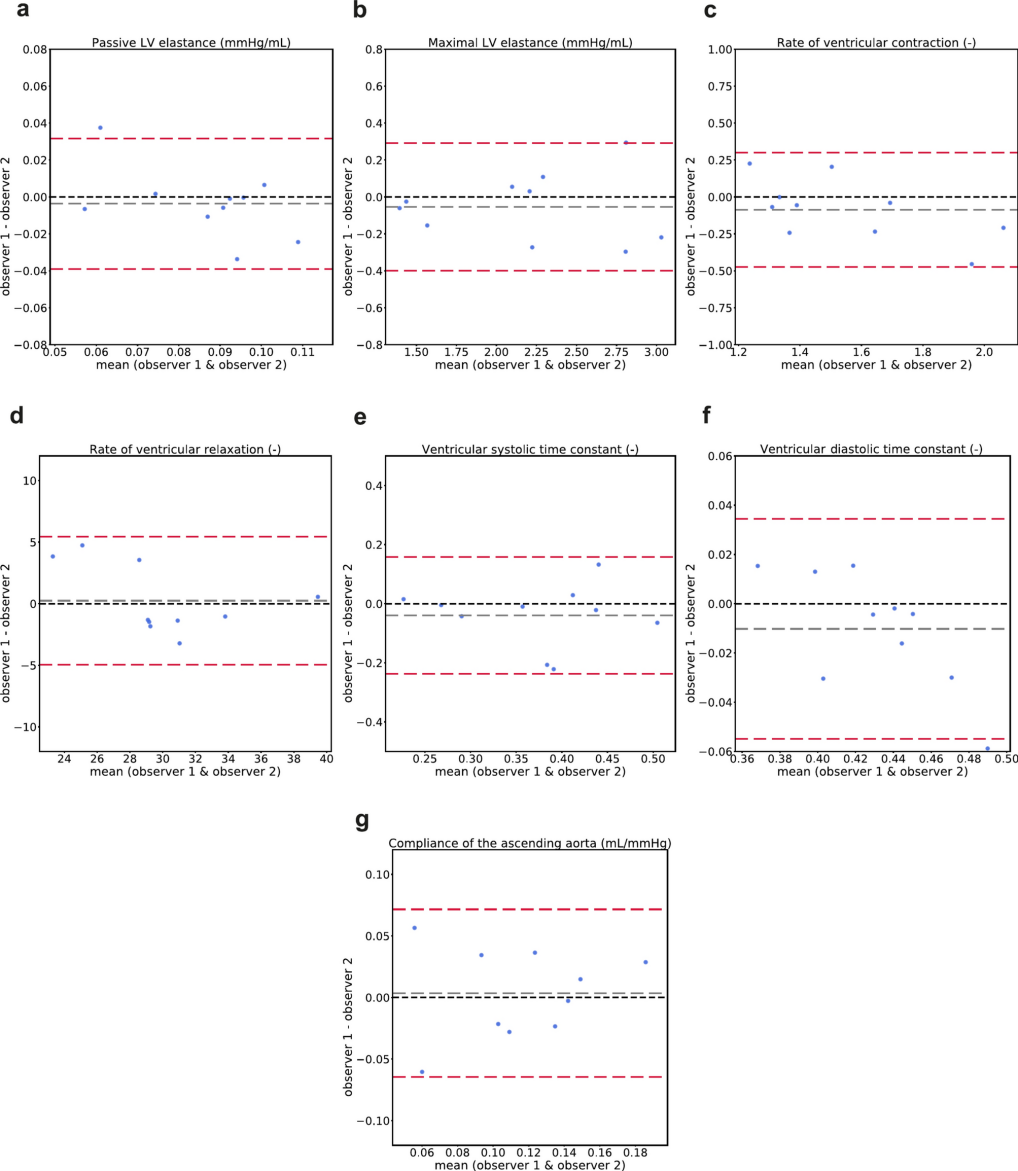
Bland–Altman plots showing agreement in the estimated model parameters using model inputs from the inter-observer variability study for **a**) passive left ventricular elastance, **b**) maximal left ventricular elastance, **c**) rate of ventricular contraction, **d**) rate of ventricular relaxation, **e**) ventricular systolic time constant, **f**) ventricular diastolic time constant, **g**) compliance of the ascending aorta. Grey dotted lines represent bias ($\overline{d}$) and red dotted lines represent limits of agreement ($\overline{d} \pm 1.96SD)$. LV: left ventricle.

Bland–Altman plots for the seven selected model-derived parameters of the left ventricular elastance and aortic compliance that were obtained with the input data from the intra- and inter-observer study are shown in Figs. 2 and 3, respectively. The mean difference values obtained from the Bland–Altman analysis, the *p*-values, and the coefficients of variation are reported in Table 2. Supplementary Tables 1 and 2 summarize the mean value, bias, and limits of agreement for all 23 personalized parameters. No significant differences were found between the mean values of estimated the parameters, computed over the analyzed subjects, in either the intra- or the inter-observer analysis (*P* >  = 0.1 in all cases, supplementary Table 1 and 2). The parameter with the lowest coefficient of variation among the selected parameters was the ventricular diastolic time constant (2.6 and 3.9% for the intra- and inter-observer analysis, respectively), while the parameter with the highest coefficient of variation when analysing both the intra- and inter-observer variability was the compliance of the ascending aorta (22 and 35%, respectively) (Table 2). The CoV in the rest of the selected parameters varied between 4.8 and 14% for the intra-observer analysis and 5.4% and 19% for the inter-observer analysis (Table 2). For all evaluated parameters in Table 2, the CoVs from the inter-observer variability analysis were slightly higher than the COVs from the corresponding intra-observer study.

**Table 2:** Repeatability of model-derived parameter estimations comparing intra- and inter- observer analysis.

Model parameter	Intra-observer			Inter-observer		
	Mean difference (− 1.96*SD, + 1.96*SD)	*P* value	CoV (%)	Mean difference (− 1.96*SD, + 1.96*SD)	*P* value	CoV (%)
Passive LV elastance (mmHg/mL)	− 0.0015 (− 0.035, 0.032)	0.91	14	− 0.0037 (− 0.041, 0.033)	0.47	17
Maximal LV elastance (mmHg/mL)	− 0.028 (− 0.29, 0.24)	0.91	4.8	− 0.054 (− 0.42, 0.31)	0.73	5.4
Rate of ventricular contraction (–)	− 0.034 (− 0.35, 0.28)	0.62	7	− 0.088 (− 0.5, 0.32)	0.57	9.3
Rate of ventricular relaxation (–)	− 0.84 (− 6.7, 5)	0.21	6.8	0.24 (− 5.2, 5.7)	0.85	7.1
Ventricular systolic time constant (–)	− 0.02 (− 0.15, 0.11)	0.62	13	− 0.04 (− 0.25, 0.17)	0.38	19
Ventricular diastolic time constant (–)	− 0.0058 (− 0.039, 0.027)	0.73	2.6	− 0.01 (− 0.057, 0.037)	0.68	3.9
Compliance of the ascending aorta (mL/mmHg)	− 0.002 (− 0.044, 0.04)	1	22	0.0035 (− 0.068, 0.075)	0.97	35

For each parameter, the mean difference and the limits of agreement ($\overline{d} \pm 1.96SD)$, the *p*-value comparing the results from the two analyses/observers, and the coefficient of variation (CoV) are presented. LV: left ventricle.

### Inter-sequence variability in model parameters

**Fig. 4:**
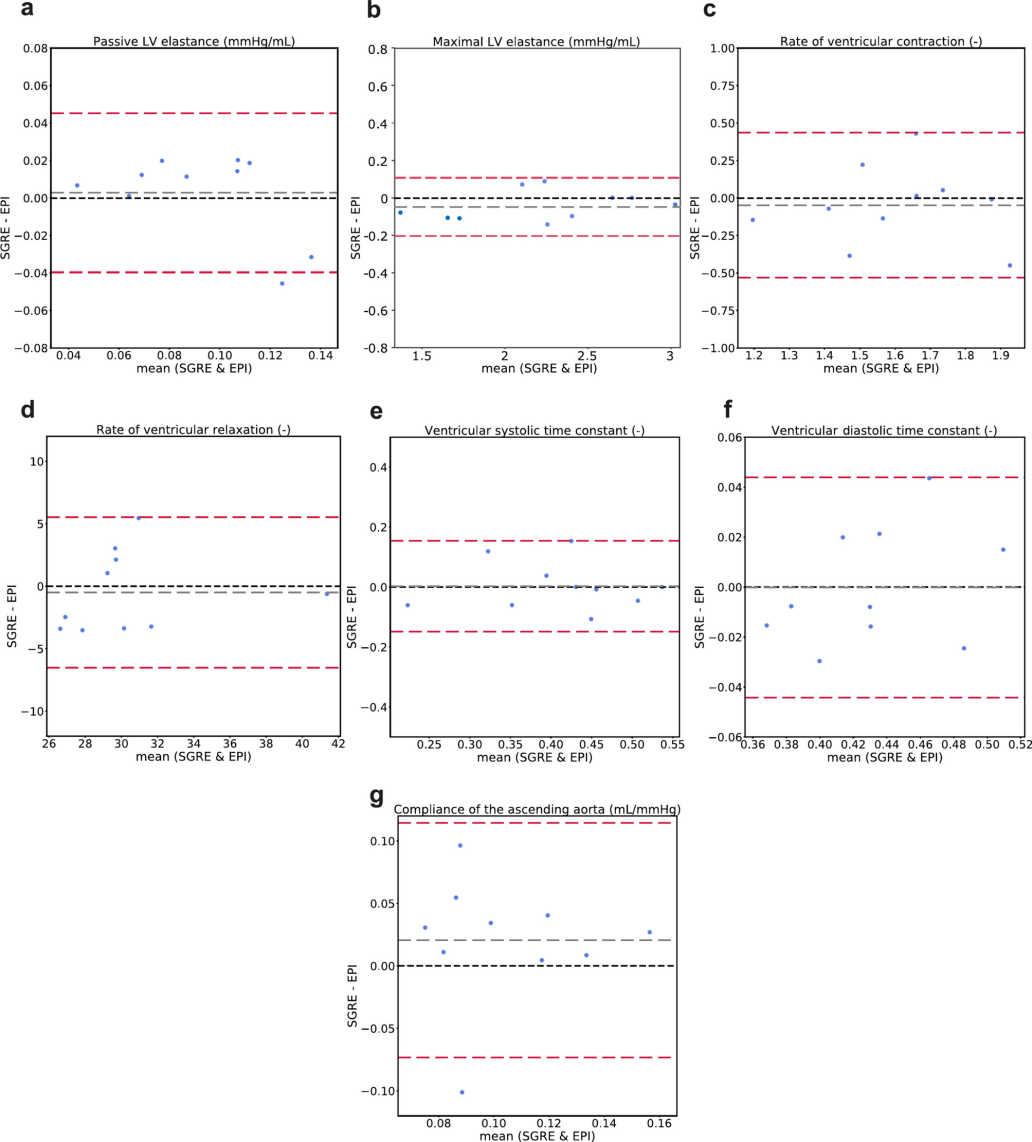
Bland–Altman plots showing agreement in the estimated model parameters using model inputs derived from the SGRE and the EPI measurements for **a**) passive left ventricular elastance, **b**) maximal left ventricular elastance, **c**) rate of ventricular contraction, **d**) rate of ventricular relaxation, **e**) ventricular systolic time constant, **f**) ventricular diastolic time constant, **g**) compliance of the ascending aorta. Grey dotted lines represent bias ($\overline{d}$) and red dotted lines represent limits of agreement ($\overline{d} \pm 1.96SD)$. LV: left ventricle.

Bland–Altman comparisons of the seven selected model parameters estimated using input data from the SGRE and EPI sequences are shown in Fig. 4. Table 3 summarizes the mean differences and limits of agreement calculated from the Bland–Altman analysis, the *p*-values, and the CoVs of the selected parameters. The corresponding results for all 23 estimated parameters, together with the mean values of the parameters, are reported in Supplementary Table 3. No significant differences were found between the values of the parameters estimated using input data from the two different sequences (*P* > 0.25 for all selected parameters, Table 3). However, one model parameter, the maximum elastance of the left atrium, had a *p*-value of 0.045 (Supplementary Table 3). The maximal LV elastance had the lowest coefficient of variation (3.3%) of the selected parameters, while the most variable parameter was the compliance of the ascending aorta (CoV 41%). For the rest of the selected model parameters, the coefficients of variance were in the range 3.6–15%.

**Table 3:** Repeatability of model-derived parameter estimations comparing results from SGRE and EPI sequences.

Model parameter	SGRE-EPI		
	Mean difference (− 1.96*SD, + 1.96*SD)	*P* value	CoV (%)
Passive LV elastance (mmHg/mL)	0.0027 (− 0.042, 0.047)	0.62	14
Maximal LV elastance (mmHg/mL)	− 0.048 (− 0.2, 0.11)	0.73	3.3
Rate of ventricular contraction (–)	− 0.048 (− 0.56, 0.46)	0.91	11
Rate of ventricular relaxation (–)	− 0.49 (− 6.8, 5.9)	1	7.5
Ventricular systolic time constant (–)	0.0031 (− 0.16, 0.16)	0.91	15
Ventricular diastolic time constant (–)	− 0.00011 (− 0.047, 0.046)	0.97	3.6
Compliance of the ascending aorta (mL/mmHg)	0.021 (− 0.078, 0.12)	0.27	41

For each parameter, the mean difference and the limits of agreement ($\overline{d} \pm 1.96SD)$, the *p*-value comparing the results from the two sequences, and the coefficient of variation (CoV) are presented. LV: left ventricle.

## Discussion

This study investigated the reproducibility of model-derived parameters describing left ventricular time-varying elastance and aortic compliance obtained using lumped-parameter modeling in combination with CMR morphological images and flow data from 4D Flow MRI^6^. The reproducibility was assessed with regard to sources of variability in the acquisition and analysis of the input measurements to the model.

Among subject-specific model parameters determining the time-varying elastance of the left ventricle, low coefficients of variation (CoV < 10%) considering both intra- and inter-observer variability were found for the maximal LV elastance, the rate of ventricular contraction, the rate of ventricular relaxation and the ventricular diastolic time constant (Table 2). The passive elastance of the left ventricle and the ventricular systolic time constant showed higher variability, with values of the coefficient of variation in the range 13–19%. The coefficients of variation for all the seven investigated model-derived parameters were higher for the inter-observer study compared to the intra-observer study (Table 2). These results are consistent with the variability found in the input measurements to the model. The inter-observer variability in these input measurements across the study subjects, in terms of both the coefficient of variation of the input parameters (Table 1) and the root mean square error of the input flow waveforms (Fig. 1D), was consistently higher than that from the intra-observer study. The association between higher variability for the inter-observer study in both the input measurements to the model and the estimated model parameters suggests that a major contribution to the variability in model parameters will be from the data analysis steps rather than the estimation approach.

Assessment of the variability in input measurements due to intra- and inter-user analysis (Table 1) involved segmentation of the left ventricle at end systole to compute the ESV, as well as calculation of the EOA at the aortic valve and the area of the LV outflow tract. The variability in ESV agree well with coefficients of variation reported in previous studies assessing intra- and inter-observer variability in LV volumes derived from CMR^20,21^. The coefficients of variation resulting from the intra- and inter-observer analysis in the EOA estimations are also in good agreement with values reported by others^22^. Similarly, the coefficients of variation found for the total flow at the mitral and aortic valves (Fig. 1) agree with those from previous studies applying retrospective valve tracking that found coefficients of variation between 3 and 7%^23^.

Comparison of model-derived parameters obtained based on the input data from the SGRE and EPI acquisitions revealed coefficients of variance comparable to those from the intra- and inter-user observer study. In comparing these sequences, the additional uncertainty in measures derived from the morphological cine MR images (i.e. calculation of the ESV) was not considered. However, the variability due to the input volumetric flow curves was higher compared to the intra- and inter-user observer studies, resulting in higher coefficients of variation for the total flows as well as higher RMSE values at the mitral valve, the aortic valve and the ascending aorta (Fig. 1). Although heart rate was not significantly different between scans, we hypothesize that these changes in heart rate and physiological changes between scans are a major cause of elevated variability in the flow waveforms, and therefore in model-derived parameters. Parameters that are sensitive to changes in heart rate, such as the ventricular contraction and relaxation rates^24,25^, may have higher variability under these circumstances, especially for large heart rate variations between sequences.

The compliance of the ascending aorta was found to be the most variable parameter in the intra- and inter-observer study and the comparison between the SGRE and EPI acquisition sequences, with coefficients of variation between 22 and 41% (Tables 2 and 3). However, the coefficient of variation for the total flow at the ascending aorta was low (2.1% to 4.2%) and similar in magnitude to the coefficients of variation in the mitral valve and the aortic valve (Fig. 1). These findings suggest that the high variability in the aortic compliance is most probably due to the relatively low temporal resolution (30–40 ms) in our study in combination with only using two aortic planes. Previous approaches to calculating pulse wave velocity (PWV) using two-dimensional phase-contrast MRI (2D PC-MRI) in two aortic locations typically require a temporal resolution lower than 11 ms^26,27^. However, 4D Flow MRI-based PWV estimation has shown to give similar results with 34–40 ms temporal resolution when utilizing all planes along the aorta^28–30^. Thus, either a higher temporal resolution or utilizing more information from the 4D flow data could improve the estimation of aortic compliance. Another potential reason for the high variability in the aortic compliance parameter is its correlation with other model parameters governing the aortic flow. A change in the aortic compliance parameter could be compensated for by changes in the aortic resistance, inductance, or the timing of the LV contraction, resulting in variability in the aortic compliance. To reduce this variability, the aortic compliance could be calculated directly from the 4D flow MRI data and used as an additional patient-specific input measurement.

Except for the reproducibility evaluated herein, there are a number of aspects of the model-derived parameters that should be further evaluated in future studies. First, the findings in this study were based on intra-modality comparisons and did not include comparisons with a gold standard method to evaluate LV time-varying elastance parameters and compliance of the ascending aorta. Future studies should investigate the variability in the model-based elastance parameters in relation to estimates based on invasively measured LV pressures and volumes recorded over a range of loading conditions and use independently measured data to validate the model predictions. Second, the sources of variability evaluated herein covers the variability resulting from a single measurement and the associated variation when changing acquisition sequence and when analysing the measured data for input to the model. However, scan-scan variability over longer periods of time or between different scanners and sites might be larger than the inter-sequence variability evaluated herein, and the change in physiological state as well as measurement uncertainty should be taken into account if comparing estimates with longer time periods in between. Third, apart from the observational uncertainty evaluated herein in point estimates of model parameter values, each parameter has an uncertainty that additionally includes factors such as model structure, physiological variation during the scan, and non-normal errors such as background phase offset in the 4D flow MRI^31^. This parameter uncertainty can be evaluated with established methods such as profile likelihood^32^. However, this requires quantification of the input data uncertainty of 4D flow MRI and cuff pressure and handling of the non-normal errors which challenges the normality assumption of the profile likelihood method. Such an analysis is not yet done, and the parameter uncertainty is therefore not included here.

Finally, only a small number of healthy subjects from a single site were included in the study. Additional studies should assess the variability in the model-derived parameters in a larger subject cohort on different scanners and with a wider spectrum of disease states, cardiac volumes, and flow characteristics as a step toward assessing the robustness of the modeling approach for clinical use. To investigate the potential effects of such larger input variability on the model output, a sensitivity analysis (see Supplementary) was performed for the input parameters of EOA at the aortic valve, the area of the LV outflow tract (A_LVOT_), and the maximum elastance of the left ventricle which depends on other inputs such as the ventricular ESV (Eq. 2). The analysis showed that variability in EOA and A_LVOT_ have small effects on the blood flow in the mitral and aortic valve and the aortic pressure (Supplementary Fig. 1). However, changes in maximum elastance had larger effects on these model outputs. This indicates that the variation in input parameters such as ESV and systolic blood pressure potentially results in larger variations in the model output and that the variation in these parameters should be kept low to maintain low variability in model output.

In conclusion, the majority of parameters derived from the modeling approach using CMR morphological images and flow data from 4D Flow MRI showed low variability with analysis and acquisition settings of the input measurements in healthy controls. Improvements in pulse wave velocity calculation and the use of automatic methods for left ventricular segmentation and analysis of the 4D Flow MRI data may reduce the uncertainty in the input measurements and thereby further decrease the variability in the model parameters. Assessment of the variability in these parameters is essential to establish the credibility of model predictions for their potential use in clinical applications, and further studies are needed to investigate variability also in patients and between different sites.

## Methods

### Study population

Ten healthy subjects (4 females, mean age 32 ± 6 years) were included in the study. All participants had no history of cardiovascular disease, no medication for cardiovascular disease and a normal physical examination. The study was approved by the Regional Ethical Review Board in Linköping and complies with the Declaration of Helsinki. All subjects provided written informed consent prior to participation in the study.

### Data acquisition

All examinations were performed using a clinical 1.5 T Philips Ingenia scanner (Philips Healthcare, Best, the Netherlands). The imaging protocol included the acquisition of morphological cine balanced steady-state free precession (bSSFP) images and two 4D Flow MRI examinations for each subject. No contrast agents were used. In addition to the acquisition of the MRI datasets, systolic and diastolic blood pressures (SBP and DBP, respectively) were obtained using cuff-based measurements in the brachial artery.

Morphological bSSFP images comprised a stack of short-axis (SA) images, as well as two-, three- and four-chamber long-axis (LAx) images of the left heart. All bSSPF images were acquired at end-expiratory breath-holds with in-plane resolution of 1 × 1 mm^2^, slice thickness of 8 mm, and acquired temporal resolution of 32–54 ms reconstructed to 30 time frames per cardiac cycle. To evaluate the effect of scan settings on the model parameters, we performed two 4D flow MRI acquisitions per subject using two different sequences: a spoiled gradient echo (SGRE) sequence with a k-space segmentation factor of 2, and an echo-planar imaging (EPI) sequence with a read-out factor of 3. The acquisitions were done in random order after each other. Both acquisitions were retrospectively cardiac gated and respiratory navigator gated and followed the recommendations from the 4D flow MRI consensus statements^31,33^. Scan parameters for both sequences were: Sagittal-oblique slab with field of view covering the whole heart and the thoracic aorta, velocity encoding (VENC) 120 cm/s and flip angle 5°, and parallel imaging (SENSE) speed up factors of 2 in the anterior–posterior (AP) and right-left (RL) directions. The echo time (TE) was 3 ms for SGRE and 4 ms for EPI, and the repetition time (TR) was 5 ms for SGRE and 7 ms for EPI. The acquired spatial resolution was 2.9 × 2.9 × 2.9 mm^3^ and the temporal resolution was 40 ms and 30 ms for the SGRE and the EPI sequence, respectively. Excluding navigator efficiency, the scan time was approximately 10 min for SGRE and 8 min for EPI. The average navigator gating efficiency was between 60 and 80%. After acquisition the 4D Flow data were retrospectively reconstructed into 40 time frames and corrected for concomitant gradient fields on the scanner. The resulting data were exported to an off-line station for post-processing and analysis. The post-processing was performed using developed software written in Matlab (The Mathworks Inc., Natick, Massachusetts, USA). The data were corrected for background errors using a weighted second-order polynomial fit to static tissue in the thorax^34^ and phase wraps using a temporal algorithm^35^.

### Data analysis

Several functional and morphological parameters were extracted from the imaging data, including parameters that characterize the morphology and the function of the left ventricle (LV) and the aortic valve, as well as volumetric flow waveforms from the heart valves and selected sites in the ascending aorta. The process of extracting parameters from data to be used to personalize the model is previously described in Casas et al. 2017 and 2018^6,13^, but is briefly set out below.

The length of the cardiac cycle was calculated as the average length of the cardiac cycle during the 4D Flow MRI acquisition. The end-systolic volume (ESV) was computed using the short-axis stack images, by manual delineation of the endocardial border at the time of end systole. The segmentations were performed using the freely available segmentation software Segment version 1.9 (Medviso, Lund, Sweden)^36^.

In the model, the aortic valve is described using the pressure gradient formulation introduced by Garcia et al.^37^. This formulation requires estimation of the effective orifice area (EOA) of the aortic valve and the cross-sectional area of the LV outflow tract (A_LVOT_), resulting in a flow-dependent description of the transvalvular pressure gradient. The EOA was assumed constant over time and calculated as follows:

1 $$EOA = SV/VTI\left( {{\text{cm}}^{2} } \right)$$

where the stroke volume (SV) was computed as the time integral of the flow at the valve, and VTI was the velocity–time integral of the instantaneous maximal velocity at the valve. A_LVOT_ was calculated as the manually delineated cross-sectional area of the LV outflow tract (LVOT), measured downstream from the valve at the timeframe of peak systole in the three chamber view^38^.

**Fig. 5:**
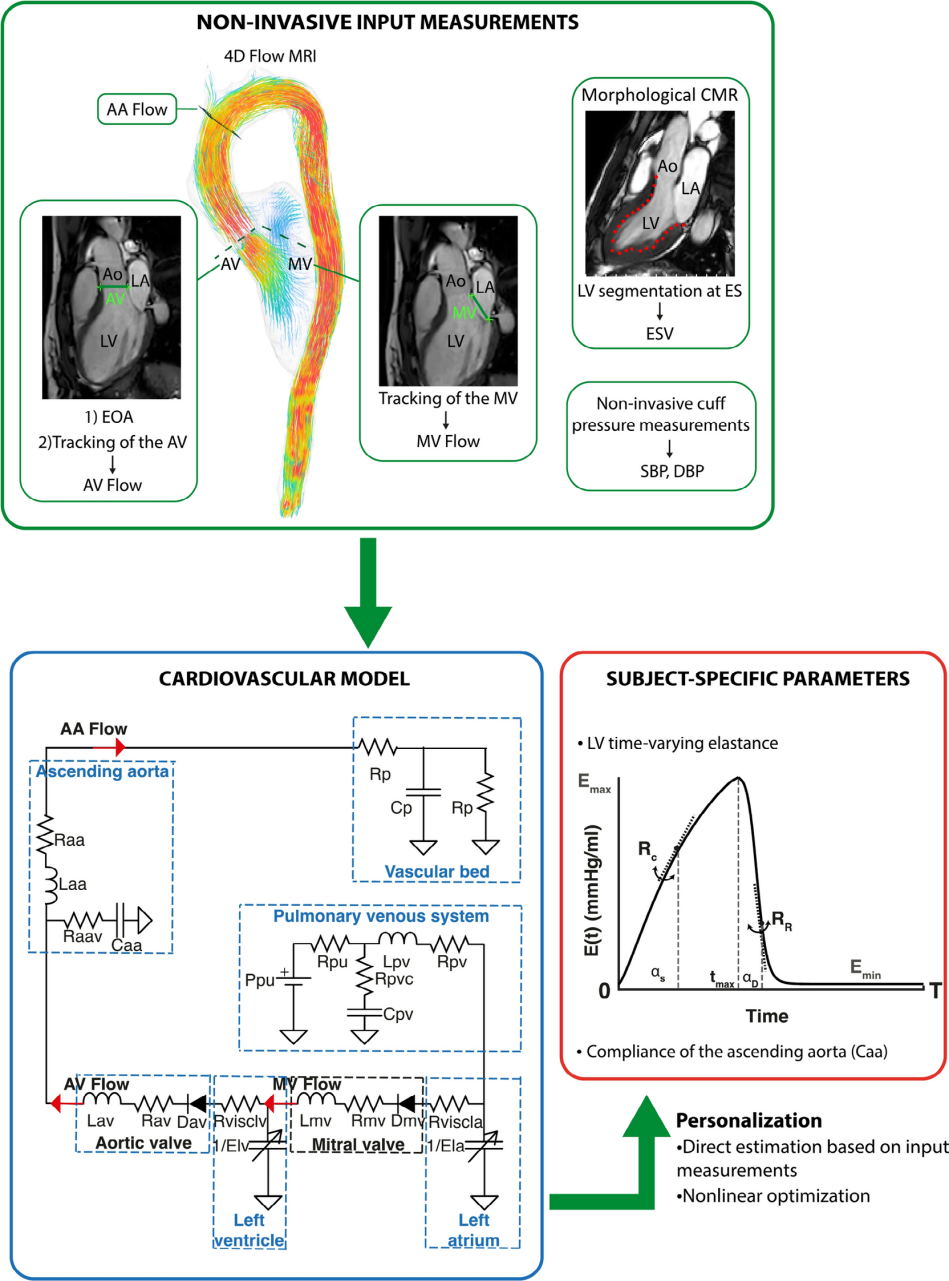
Schematic diagram of the personalization approach. The lumped parameter model is personalized using input measurements derived from the 4D Flow MRI data, the morphological CMR images and the non-invasive cuff pressure measurements in the brachial artery (SBP and DBP). Personalization yields subject-specific values of the model parameters, including the compliance of the ascending aorta (Caa) and parameters describing the LV time-varying elastance. The flow waveforms for personalization include the volumetric flows across the mitral valve (MV Flow), the aortic valve (AV Flow) and the ascending aorta, upstream from the brachiocephalic trunk (AA Flow). The time-varying elastance over the cardiac cycle length (T) is determined by the end-systolic, maximal elastance ($$E_{max}$$), the minimal elastance ($$E_{min} )$$ over the duration of the cardiac cycle (T), the rates of LV contraction $$(R_{C} )$$ and relaxation ($$R_{R}$$), and the systolic and diastolic time constants $$(\alpha_{s}$$ and $$\alpha_{D}$$, respectively). The maximal elastance occurs at the time of end-systole ($$t_{max} )$$. A description of the parameters in the model is given in Casas et al.^6^. Ao, aorta; ES, End systole; EOA, effective orifice area; LA, left atrium; LV, left ventricle.

Input flow waveforms to the model include 4D Flow MRI-derived time-resolved volumetric flow waveforms at the mitral valve, the aortic valve, and the ascending aorta, upstream from the brachiocephalic trunk. Flows through the heart valves were computed using a semi-automatic, retrospective valve tracking approach with correction for through-plane motion. Using this approach, two input points that identify the valve annulus are manually placed in the three-chamber view at the time of end diastole. By automatic tracking of the valve annulus along the successive time frames, in combination with manual segmentation of the valve orifice for each time frame, the approach yields time-resolved curves of the flow through the valve. These calculations were performed using in-house software written in Matlab (The Mathworks Inc., Natick, Massachusetts, USA). To extract the flow waveforms in the ascending aorta, a 4D PC-MR angiogram was used for anatomical orientation in 3D using commercially available visualization software (EnSight, CEI Inc., NC, USA) to locate the position of the analysis plane. The volumetric flow curves were calculated by manual segmentation of the aortic contour for every cardiac time frame, as described elsewhere^6^. A schematic summary of the steps involved in the analysis process and the modeling approach is provided in Fig. 5.

### Subject-specific parameter estimation

The model comprises 40 parameters, of which 23 were tuned to be subject-specific. The remaining 17 parameters were set to values reported in previous studies^3,39–42^. The subject-specific parameters were obtained either by direct estimation from the input measurements or using a nonlinear optimization approach based on minimizing the error between the model-generated flow waveforms and those extracted from the 4D Flow MRI datasets. The input parameter T, length of cardiac cycle, was directly estimated from the measurements. The parameters effective orifice area of the aortic valve (EOA), left ventricular outflow tract area (A_LVOT_), and ventricular elastance (EmaxLV) were estimated from measurements but allowed to vary ± 10% during optimization. EmaxLV was calculated as

2 $$\begin{gathered} Emax_{LV} = 0.9*\frac{{SBP + Pressure\;gradient_{max} }}{{ESV - V0_{LV} }}, \hfill \\ Pressure\;gradient_{max} = \frac{\rho }{{2 ELCo^{2} }}*\max \left( {AV_{Flow} } \right)^{2} *\left( {\frac{0.06}{{133.322}}} \right) \hfill \\ \end{gathered}$$

where $AV_{Flow}$ is the blood flow through the aortic valve, SBP is the measured brachial systolic pressure, $\rho$ is the density of blood set to 1.06 g/mL, ESV is the measured end systolic stroke volume of the left ventricle, and $V0_{LV}$ is the unstressed blood volume in the left ventricle, and ELCo is the energy loss coefficient defined as (EOA* A_LVOT_)/(A_LVOT_ − EOA). The remaining 20 parameters, including aortic compliance and the remaining parameters governing the left ventricular elastance, were estimated using the nonlinear optimization routine. Details of the cardiovascular model and the optimization process are given in Casas et al.^6,13^.

### Intra- and inter-observer analysis and inter-sequence variability

The datasets were analyzed by two observers with four years of experience in cardiovascular MR. To evaluate the intra-observer variability, one observer (BC) performed two blinded analyses of the morphological and 4D Flow images for the ten study subjects, with approximately one month in between. The analysis included computation of the ESV, the EOA, the A_LVOT_, and the volumetric flow waveforms at the mitral valve, the aortic valve, and the ascending aorta. Inter-observer analysis was performed independently by a second observer (FV) who repeated the analysis on the same set of images. For the inter-scan variability, the second observer (FV) analyzed the 4D Flow MRI datasets acquired with the SGRE and the EPI sequences for the ten subjects in the study. This analysis involved calculation of the EOA, A_LVOT_, and the flow waveforms at the mitral valve, the aortic valve, and the ascending aorta. The input data derived from the intra-observer, inter-observer, and inter-sequence analyses were used as input to estimate the subject-specific model parameters and evaluate their variability.

### Statistical analysis

Statistical analysis was performed using Matlab (ver. R2023a, the Mathworks, Inc., Natick, Massachusetts, USA). All data are reported as mean ± standard deviation (SD) unless otherwise stated. Wilcoxon rank sum tests were performed for comparing image-derived measurements and model parameters obtained in the intra- and inter-observer studies, as well as from different sequences. A *P* value < 0.05 was considered significant. The reproducibility of the results was evaluated using the coefficient of variation (CoV)^43^. In addition, Bland–Altman plots were used to visually assess the agreement between the results. The bias $\overline{d }$ and the limits of agreement ($\overline{d } \pm 1.96SD)$ were derived from the Bland–Altman analysis.

## Supplementary Information

Supplementary Information.

## Data Availability

The MRI datasets used to construct the models are available from the Linköping University Hospital, upon request to the corresponding author, for researchers who meet the criteria for access to confidential data. The codes to perform the statistical analysis and the sensitivity analysis are provided at https://github.com/kajtu/input_variability and Zenodo^44^.

## References

[1] Gray, R. A. & Pathmanathan, P. Patient-specific cardiovascular computational modeling: Diversity of personalization and challenges. *J. Cardiovasc. Trans. Res.* **11**, 80–88 (2018). 10.1007/s12265-018-9792-2 PMC5908828 29512059

[2] Shi, Y., Lawford, P. & Hose, R. Review of zero-D and 1-D models of blood flow in the cardiovascular system. *Biomed. Eng.* **10**, 33 (2011). 10.1186/1475-925X-10-33 PMC3103466 21521508

[3] Sun, Y., Sjoberg, B. J., Ask, P., Loyd, D. & Wranne, B. Mathematical model that characterizes transmitral and pulmonary venous flow velocity patterns. *Am. J. Physiol.* **268**, H476–H489 (1995). 7840296 10.1152/ajpheart.1995.268.1.H476

[4] Colunga, A. L., Colebank, M. J. & Olufsen, M. S. Parameter inference in a computational model of haemodynamics in pulmonary hypertension. *J. R. Soc. Interface* **20**, 20220735 (2023). 36854380 10.1098/rsif.2022.0735 PMC9974303

[5] Pant, S. et al. Data assimilation and modelling of patient-specific single-ventricle physiology with and without valve regurgitation. *J. Biomech.* **49**, 2162–2173 (2016). 26708918 10.1016/j.jbiomech.2015.11.030

[6] Casas, B. et al. Bridging the gap between measurements and modelling: A cardiovascular functional avatar. *Sci. Rep.* **7**, 6214 (2017). 28740184 10.1038/s41598-017-06339-0 PMC5524911

[7] Keshavarz-Motamed, Z. et al. Non-invasive determination of left ventricular workload in patients with aortic stenosis using magnetic resonance imaging and doppler echocardiography. *PLoS One* **9**, e86793 (2014). 24489786 10.1371/journal.pone.0086793 PMC3904946

[8] Garcia-Canadilla, P. et al. Patient-specific estimates of vascular and placental properties in growth-restricted fetuses based on a model of the fetal circulation. *Placenta* **36**, 981–989 (2015). 26242709 10.1016/j.placenta.2015.07.130

[9] Tunedal, K. et al. Haemodynamic effects of hypertension and type 2 diabetes: Insights from a 4D flow MRI-based personalized cardiovascular mathematical model. *J. Physiol.* **601**, 3765–3787 (2023). 37485733 10.1113/JP284652

[10] Harrod, K. K., Rogers, J. L., Feinstein, J. A., Marsden, A. L. & Schiavazzi, D. E. Predictive modeling of secondary pulmonary hypertension in left ventricular diastolic dysfunction. *Front. Physiol.*10.3389/fphys.2021.666915 (2021). 34276397 10.3389/fphys.2021.666915 PMC8281259

[11] Jones, E. et al. Phenotyping heart failure using model-based analysis and physiology-informed machine learning. *J. Physiol.* **599**, 4991–5013 (2021). 34510457 10.1113/JP281845 PMC8595692

[12] Garber, L., Khodaei, S. & Keshavarz-Motamed, Z. The critical role of lumped parameter models in patient-specific cardiovascular simulations. *Arch. Computat. Methods Eng.* **29**, 2977–3000 (2022).

[13] Casas, B. et al. Non-invasive assessment of systolic and diastolic cardiac function during rest and stress conditions using an integrated image-modeling approach. *Front. Physiol.* **9**, 1515 (2018). 30425650 10.3389/fphys.2018.01515 PMC6218619

[14] Colijn, C., Jones, N., Johnston, I. G., Yaliraki, S. & Barahona, M. Toward precision healthcare: Context and mathematical challenges. *Front. Physiol.* **8**, 136 (2017). 28377724 10.3389/fphys.2017.00136 PMC5359292

[15] Mirams, G. R., Pathmanathan, P., Gray, R. A., Challenor, P. & Clayton, R. H. Uncertainty and variability in computational and mathematical models of cardiac physiology. *J. Physiol.* **594**, 6833–6847 (2016). 26990229 10.1113/JP271671 PMC5134370

[16] Vernon, I., Goldstein, M. & Bower, R. G. Galaxy formation: A Bayesian uncertainty analysis. *Bayesian Anal.* **5**, 619–669 (2010).

[17] Kennedy, M. C. & O’Hagan, A. Bayesian calibration of computer models. *J. R. Stat. Soc. Ser. B (Stat. Methodol.)* **63**, 425–464 (2001).

[18] Ninos, G., Bartzis, V., Merlemis, N. & Sarris, I. E. Uncertainty quantification implementations in human hemodynamic flows. *Comput. Methods Progr. Biomed.* **203**, 106021 (2021). 10.1016/j.cmpb.2021.106021 33721602

[19] Suga, H., Sagawa, K. & Shoukas, A. A. Load independence of the instantaneous pressure-volume ratio of the canine left ventricle and effects of epinephrine and heart rate on the ratio. *Circ. Res.* **32**, 314–322 (1973). 4691336 10.1161/01.res.32.3.314

[20] Bogaert, J. G. et al. Left ventricular quantification with breath-hold MR imaging: Comparison with echocardiography. *MAGMA* **3**, 5–12 (1995). 7600177 10.1007/BF02426395

[21] Stoll, V. M. et al. Test-retest variability of left ventricular 4D flow cardiovascular magnetic resonance measurements in healthy subjects. *J. Cardiovasc. Magn. Reson.* **20**, 15 (2018). 29499706 10.1186/s12968-018-0432-4 PMC5833126

[22] Garcia, J. et al. Evaluation of aortic stenosis severity using 4D flow jet shear layer detection for the measurement of valve effective orifice area. *Magn. Reson. Imaging* **32**, 891–898 (2014). 24865143 10.1016/j.mri.2014.04.017 PMC4099275

[23] Roes, S. D. et al. Flow assessment through four heart valves simultaneously using 3-dimensional 3-directional velocity-encoded magnetic resonance imaging with retrospective valve tracking in healthy volunteers and patients with valvular regurgitation. *Invest. Radiol.* **44**, 669–675 (2009). 19724233 10.1097/RLI.0b013e3181ae99b5

[24] Kilner, P. J., Henein, M. Y. & Gibson, D. G. Our tortuous heart in dynamic mode—An echocardiographic study of mitral flow and movement in exercising subjects. *Heart Vessels* **12**, 103–110 (1997). 9496460 10.1007/BF02767127

[25] Paelinck, B. P., Lamb, H. J., Bax, J. J., van der Wall, E. E. & de Roos, A. MR flow mapping of dobutamine-induced changes in diastolic heart function. *J. Magn. Reson. Imaging* **19**, 176–181 (2004). 14745750 10.1002/jmri.10448

[26] Fielden, S. W. et al. A new method for the determination of aortic pulse wave velocity using cross-correlation on 2D PCMR velocity data. *J. Magn. Reson. Imaging* **27**, 1382–1387 (2008). 18504758 10.1002/jmri.21387

[27] Hsi-Yu, Y., Hsu-Hsia, P., Jaw-Lin, W., Chih-Yung, W. & Isaac, T.W.-Y. Quantification of the pulse wave velocity of the descending aorta using axial velocity profiles from phase-contrast magnetic resonance imaging. *Magn. Reson. Med.* **56**, 876–883 (2006). 16947380 10.1002/mrm.21034

[28] Nguyen, L.-A. et al. Aortic stiffness measured from either 2D/4D flow and cine MRI or applanation tonometry in coronary artery disease: A case-control study. *J. Clin. Med.* **12**, 3643 (2023). 37297837 10.3390/jcm12113643 PMC10253575

[29] Lindenberger, M., Ziegler, M., Bjarnegård, N., Ebbers, T. & Dyverfeldt, P. Regional and global aortic pulse wave velocity in patients with abdominal aortic aneurysm. *Eur. J. Vasc. Endovasc. Surg.* **67**, 506–513 (2024). 37777048 10.1016/j.ejvs.2023.09.040

[30] Dyverfeldt, P., Ebbers, T. & Länne, T. Pulse wave velocity with 4D flow MRI: Systematic differences and age-related regional vascular stiffness. *Magn. Reson. Imaging* **32**, 1266–1271 (2014). 25171817 10.1016/j.mri.2014.08.021

[31] Dyverfeldt, P. et al. 4D flow cardiovascular magnetic resonance consensus statement. *J. Cardiovasc. Magn. Reson.* **17**, 72 (2015). 26257141 10.1186/s12968-015-0174-5 PMC4530492

[32] Kreutz, C., Raue, A., Kaschek, D. & Timmer, J. Profile likelihood in systems biology. *FEBS J.* **280**, 2564–2571 (2013). 23581573 10.1111/febs.12276

[33] Bissell, M. M. et al. 4D Flow cardiovascular magnetic resonance consensus statement: 2023 update. *J. Cardiovasc. Magn. Reson.* **25**, 40 (2023). 37474977 10.1186/s12968-023-00942-z PMC10357639

[34] Ebbers, T. *et al.* Higher order weighted least-squares phase offset correction for improved accuracy in phase-contrast MRI. In *Proceedings ISMRM* (Toronto, Canada, 2008).

[35] Xiang, Q.-S. Temporal phase unwrapping for cine velocity imaging. *J. Magn. Reson. Imaging* **5**, 529–534 (1995). 8574036 10.1002/jmri.1880050509

[36] Heiberg, E. et al. Design and validation of segment—Freely available software for cardiovascular image analysis. *BMC Med. Imaging*10.1186/1471-2342-10-1 (2010). 20064248 10.1186/1471-2342-10-1 PMC2822815

[37] Garcia, D., Pibarot, P. & Durand, L.-G. Analytical modeling of the instantaneous pressure gradient across the aortic valve. *J. Biomech.* **38**, 1303–1311 (2005). 15863115 10.1016/j.jbiomech.2004.06.018

[38] Garcia, J., Kadem, L., Larose, E., Clavel, M.-A. & Pibarot, P. Comparison between cardiovascular magnetic resonance and transthoracic Doppler echocardiography for the estimation of effective orifice area in aortic stenosis. *J. Cardiovasc. Magn. Reson.* **13**, 25 (2011). 21527021 10.1186/1532-429X-13-25 PMC3108925

[39] Heldt, T., Shim, E. B., Kamm, R. D. & Mark, R. G. Computational modeling of cardiovascular response to orthostatic stress. *J. Appl. Physiol.* **92**, 1239–1254 (2002). 11842064 10.1152/japplphysiol.00241.2001

[40] Garcia, J. et al. Cardiovascular magnetic resonance evaluation of aortic stenosis severity using single plane measurement of effective orifice area. *J. Cardiovasc. Magn. Reson.* **14**, 23 (2012). 22480269 10.1186/1532-429X-14-23 PMC3366866

[41] Mynard, J. P., Davidson, M. R., Penny, D. J. & Smolich, J. J. A simple, versatile valve model for use in lumped parameter and one-dimensional cardiovascular models. *Int. J. Numer. Methods Biomed. Eng.* **28**, 626–641 (2012). 10.1002/cnm.1466 25364842

[42] Broomé, M., Maksuti, E., Bjällmark, A., Frenckner, B. & Janerot-Sjöberg, B. Closed-loop real-time simulation model of hemodynamics and oxygen transport in the cardiovascular system. *BioMed. Eng.* **12**, 69 (2013). 10.1186/1475-925X-12-69 PMC3751725 23842033

[43] Hyslop, N. P. & White, W. H. Estimating precision using duplicate measurements. *J. Air Waste Manag. Assoc.* **59**, 1032–1039 (2009). 19785269 10.3155/1047-3289.59.9.1032

[44] Tunedal, K. & Garcia, C. Belén. Input variability code. Zenodo 10.5281/zenodo.14152522 (2024).

