# Supplementary material for: Observer- and sequence variability in personalized 4D flow MRI-based cardiovascular models
Source: Sci Rep. 2025 Jan 8;15:1352. doi: 10.1038/s41598-024-84390-4 (PMC11711780; doi:10.1038/s41598-024-84390-4)
Supplement: Supplementary file 1 — Supplementary Information. [file 41598_2024_84390_MOESM1_ESM.pdf]

SUPPLEMENTARY MATERIAL

Observer- and sequence variability in personalized 4D flow MRI-based cardiovascular models

Belén Casas Garcia[1,2], Kajsa Tunedal[2,3], Federica Viola[1], Gunnar Cedersund[2,3,4],

Carl-Johan Carlhäll[1,2,5], Matts Karlsson[2,6], Tino Ebbers*[1,2]

This supplementary material contains results of intra- and inter-observer and inter-sequence variability for all estimated model parameters and measured total blood flow volumes, and a sensitivity analysis of the effects of changing some of the input parameters.

Code for the analysis is provided at: https://github.com/kajtu/input_variability

1. Department of Health, Medicine and Caring Sciences, Linköping University, Linköping, Sweden.

2. Center for Medical Image Science and Visualization (CMIV), Linköping University, Linköping, Sweden.

3. Department of Biomedical Engineering, Linköping University, Linköping, Sweden.

4. School of Medical Sciences and Inflammatory Response and Infection Susceptibility Centre (iRiSC), Faculty of Medicine and Health, Örebro University, Örebro, Sweden

5. Department of Clinical Physiology in Linköping, and Department of Health, Medicine and Caring Sciences, Linköping University, Linköping, Sweden.

6. Department of Management and Engineering, Linköping University, Linköping, Sweden.

*Corresponding author
Tino Ebbers, Department of Health, Medicine and Caring Sciences,
Linköping University,
SE-581 83 Linköping, Sweden.
E-mail: tino.ebbers@liu.se

Intra- and inter-observer and inter-sequence variability for all estimated model parameters and measured blood flow

Table 1: Intra-observer variability for all model-based parameters. The bias $\bar{d}$ and the limits of agreement ($\bar{d} \pm 1.96SD$) were derived from the Bland-Altman analysis. The p-value, coefficient of variation (**CoV**), and mean value and standard deviation (**SD**) of all subjects and both analyses are also presented for each parameter. A_{LVOT}: left ventricular outflow tract area, Caa: aortic compliance, EOA: effective orifice area of the aortic valve, Emax_LA: maximal elastance of the LA, Emax_LV: maximal elastance of the LV, Emin_LA: minimal (passive) elastance of the LA, Emin_LV: minimal (passive) elastance of the LV, Lao: inertance of the ascending aorta, Lav: inertance of the aortic valve, Lmv: inertance of the mitral valve, Ppu: pulmonary capillary pressure, Rao: resistance of the ascending aorta, Rmv: resistance of the mitral valve, k_diast_LA: diastolic time constant of the left atrium, k_diast_LV: diastolic time constant of the left ventricle, k_syst_LA: systolic time constant of the left atrium, k_syst_LV: systolic time constant of the left ventricle, m1_LA: contraction rate of the left atrium, m1_LV: contraction rate of the left ventricle, m2_LA: relaxation rate of the left atrium, m2_LV: relaxation rate of the left ventricle, onset_LA: onset of the contraction of the left atrium, onset_LV: onset of the contraction of the left ventricle, T: length of cardiac cycle.

**Parameter**	**Unit**	**Descriptive Statistics** *Mean ± SD*	**Limits of agreement** $\bar{d}$ *(-1.96*SD, +1.96*SD)*	**P value**	**CoV (%)**
A_{LVOT}	cm^2	6.9 ± 1.2	-0.17 (-0.69, 0.35)	0.73	2.9
Caa	mL/mmHg	0.12 ± 0.043	-0.002 (-0.044, 0.04)	1	22
EOA	cm^2	3.1 ± 0.46	0.05 (-0.084, 0.18)	0.68	1.5
Emax_LA	mmHg/mL	0.16 ± 0.038	-0.025 (-0.099, 0.049)	0.14	18
Emax_LV	mmHg/mL	2.2 ± 0.54	-0.028 (-0.29, 0.24)	0.91	4.8
Emin_LA	mmHg/mL	0.11 ± 0.023	-0.0079 (-0.048, 0.032)	0.43	12
Emin_LV	mmHg/mL	0.085 ± 0.02	-0.0015 (-0.035, 0.032)	0.91	14
Lao	mmHg*s^2/mL	0.00019 ± 0.00013	-7.6e-06 (-0.00024, 0.00023)	0.34	31
Lav	mmHg*s^2/mL	0.00016 ± 8.6e-05	-2.4e-05 (-0.00016, 0.00011)	0.62	35
Lmv	mmHg*s^2/mL	0.00056 ± 0.00019	-5.2e-05 (-0.00038, 0.00027)	0.47	17
Ppu	mmHg	9.4 ± 1.7	-0.17 (-2.7, 2.3)	0.68	8.6
Rao	mmHg·s/mL	0.075 ± 0.0076	-0.0012 (-0.0035, 0.001)	0.24	1.5
Rmv	mmHg·s/mL	0.0047 ± 0.0012	-0.00022 (-0.0016, 0.0012)	0.97	11
k_diast_LA	s	0.18 ± 0.052	-0.014 (-0.13, 0.1)	0.85	16
k_diast_LV	s	0.43 ± 0.035	-0.0058 (-0.039, 0.027)	0.73	2.6
k_syst_LA	s	0.098 ± 0.042	0.0011 (-0.1, 0.11)	0.57	31
k_syst_LV	s	0.36 ± 0.098	-0.02 (-0.15, 0.11)	0.62	13
m1_LA	-	1.2 ± 0.55	-0.27 (-1.6, 1.1)	0.85	32
m1_LV	-	1.5 ± 0.22	-0.034 (-0.35, 0.28)	0.62	7
m2_LA	-	16 ± 6.1	-0.28 (-6.2, 5.6)	1	15
m2_LV	-	31 ± 3.1	-0.84 (-6.7, 5)	0.21	6.8
onset_LA	Fraction of T	0.81 ± 0.08	-0.046 (-0.34, 0.25)	0.52	14
onset_LV	Fraction of T	-0.047 ± 0.025	0.0078 (-0.038, 0.053)	0.68	61

Table 2: Inter-observer variability for all model-based parameters. The bias $\bar{d}$ and the limits of agreement ($\bar{d} \pm 1.96SD$) were derived from the Bland-Altman analysis. The p-value, coefficient of variation (CoV), and mean value and standard deviation (SD) of all subjects and both observers are also presented for each parameter. A_{LVOT}: left ventricular outflow tract area, Caa: aortic compliance, EOA: effective orifice area of the aortic valve, Emax_LA: maximal elastance of the LA, Emax_LV: maximal elastance of the LV, Emin_LA: minimal (passive) elastance of the LA, Emin_LV: minimal (passive) elastance of the LV, Lao: inertance of the ascending aorta, Lav: inertance of the aortic valve, Lmv: inertance of the mitral valve, Ppu: pulmonary capillary pressure, Rao: resistance of the ascending aorta, Rmv: resistance of the mitral valve, k_diast_LA: diastolic time constant of the left atrium, k_diast_LV: diastolic time constant of the left ventricle, k_syst_LA: systolic time constant of the left atrium, k_syst_LV: systolic time constant of the left ventricle, m1_LA: contraction rate of the left atrium, m1_LV: contraction rate of the left ventricle, m2_LA: relaxation rate of the left atrium, m2_LV: relaxation rate of the left ventricle, onset_LA: onset of the contraction of the left atrium, onset_LV: onset of the contraction of the left ventricle, T: length of cardiac cycle.

**Parameter**	**Unit**	**Descriptive Statistics** ***Mean ± SD***	**Limits of agreement** $\bar{d}$ ***(-1.96*SD, +1.96*SD)***	**P value**	**CoV (%)**
A_{LVOT}	cm^2	6.9 ± 1.5	-0.15 (-3, 2.7)	0.97	13
Caa	mL/mmHg	0.12 ± 0.043	0.0035 (-0.068, 0.075)	0.97	35
EOA	cm^2	3.2 ± 0.55	-0.21 (-0.9, 0.49)	0.27	8.4
Emax_LA	mmHg/mL	0.16 ± 0.045	-0.031 (-0.1, 0.041)	0.1	18
Emax_LV	mmHg/mL	2.2 ± 0.58	-0.054 (-0.42, 0.31)	0.73	5.4
Emin_LA	mmHg/mL	0.11 ± 0.021	-0.0089 (-0.033, 0.015)	0.34	10
Emin_LV	mmHg/mL	0.086 ± 0.019	-0.0037 (-0.041, 0.033)	0.47	17
Lao	mmHg*s^2/mL	0.00022 ± 0.0002	-6.5e-05 (-0.0005, 0.00037)	0.34	38
Lav	mmHg*s^2/mL	0.00016 ± 9e-05	-1.7e-05 (-0.00016, 0.00012)	0.47	32
Lmv	mmHg*s^2/mL	0.00054 ± 0.00017	-9.7e-06 (-0.00027, 0.00025)	0.85	16
Ppu	mmHg	9.4 ± 1.8	-0.089 (-3.7, 3.5)	0.97	12
Rao	mmHg·s/mL	0.075 ± 0.0072	-0.0019 (-0.0084, 0.0047)	0.47	3.6
Rmv	mmHg·s/mL	0.0045 ± 0.0014	0.00018 (-0.0014, 0.0017)	0.62	13
k_diast_LA	s	0.19 ± 0.05	-0.034 (-0.13, 0.066)	0.19	20
k_diast_LV	s	0.43 ± 0.037	-0.01 (-0.057, 0.037)	0.68	3.9
k_syst_LA	s	0.11 ± 0.047	-0.02 (-0.12, 0.082)	0.52	28
k_syst_LV	s	0.37 ± 0.1	-0.04 (-0.25, 0.17)	0.38	19
m1_LA	-	1.1 ± 0.43	-0.12 (-0.97, 0.74)	0.97	26
m1_LV	-	1.5 ± 0.3	-0.088 (-0.5, 0.32)	0.57	9.3
m2_LA	-	15 ± 5.2	0.49 (-11, 12)	1	25
m2_LV	-	30 ± 4.6	0.24 (-5.2, 5.7)	0.85	7.1
onset_LA	Fraction of T	0.79 ± 0.08	-0.0031 (-0.057, 0.051)	0.91	3
onset_LV	Fraction of T	-0.05 ± 0.029	0.013 (-0.041, 0.066)	0.38	63

Table 3: Inter-sequence variability for all model-based parameters. The bias $\bar{d}$ and the limits of agreement ($\bar{d} \pm 1.96SD$) were derived from the Bland-Altman analysis. The p-value, coefficient of variation (CoV), and mean value and standard deviation (SD) of all subjects and both sequences are also presented for each parameter. A_{LVOT}**:** left ventricular outflow tract area**,** Caa: aortic compliance, EOA: effective orifice area of the aortic valve, Emax_LA: maximal elastance of the LA, Emax_LV: maximal elastance of the LV, Emin_LA: minimal (passive) elastance of the LA, Emin_LV: minimal (passive) elastance of the LV, Lao: inertance of the ascending aorta, Lav: inertance of the aortic valve, Lmv: inertance of the mitral valve, Ppu: pulmonary capillary pressure, Rao: resistance of the ascending aorta, Rmv: resistance of the mitral valve, k_diast_LA: diastolic time constant of the left atrium, k_diast_LV: diastolic time constant of the left ventricle, k_syst_LA: systolic time constant of the left atrium, k_syst_LV: systolic time constant of the left ventricle, m1_LA: contraction rate of the left atrium, m1_LV: contraction rate of the left ventricle, m2_LA: relaxation rate of the left atrium, m2_LV: relaxation rate of the left ventricle, onset_LA: onset of the contraction of the left atrium, onset_LV: onset of the contraction of the left ventricle, T: length of cardiac cycle.

**Parameter**	**Unit**	**Descriptive Statistics** ***Mean ± SD***	**Limits of agreement** ***$\bar{d}$ (-1.96*SD, +1.96*SD)***	**P value**	**CoV (%)**
A_{LVOT}	cm^2	7 ± 1.7	0.48 (-1.5, 2.4)	0.62	9.2
Caa	mL/mmHg	0.1 ± 0.037	0.021 (-0.078, 0.12)	0.27	41
EOA	cm^2	3.2 ± 0.65	0.083 (-0.64, 0.81)	0.57	8.1
Emax_LA	mmHg/mL	0.15 ± 0.031	0.025 (-0.052, 0.1)	0.045	21
Emax_LV	mmHg/mL	2.2 ± 0.51	-0.048 (-0.2, 0.11)	0.73	3.3
Emin_LA	mmHg/mL	0.1 ± 0.023	0.02 (-0.012, 0.053)	0.054	17
Emin_LV	mmHg/mL	0.093 ± 0.031	0.0027 (-0.042, 0.047)	0.62	14
Lao	mmHg*s^2/mL	0.00012 ± 4.5e-05	1.5e-05 (-0.00013, 0.00016)	0.91	31
Lav	mmHg*s^2/mL	0.00014 ± 0.00012	-1.6e-05 (-0.00035, 0.00032)	0.38	42
Lmv	mmHg*s^2/mL	0.00061 ± 0.00016	5.8e-05 (-0.00018, 0.0003)	0.31	13
Ppu	mmHg	9.7 ± 1.4	0.58 (-3.1, 4.3)	0.34	14
Rao	mmHg·s/mL	0.079 ± 0.00087	0.0006 (-0.0016, 0.0028)	0.24	1.1
Rmv	mmHg·s/mL	0.0052 ± 0.0016	-0.00062 (-0.0026, 0.0013)	0.43	17
k_diast_LA	s	0.17 ± 0.044	0.0019 (-0.11, 0.12)	0.68	22
k_diast_LV	s	0.43 ± 0.045	-0.00011 (-0.047, 0.046)	0.97	3.6
k_syst_LA	s	0.099 ± 0.043	0.0053 (-0.054, 0.065)	1	17
k_syst_LV	s	0.41 ± 0.098	0.0031 (-0.16, 0.16)	0.91	15
m1_LA	-	1.1 ± 0.35	-0.28 (-1.1, 0.58)	0.12	31
m1_LV	-	1.6 ± 0.25	-0.048 (-0.56, 0.46)	0.91	11
m2_LA	-	13 ± 5.5	0.67 (-4.7, 6)	0.79	19
m2_LV	-	30 ± 4.4	-0.49 (-6.8, 5.9)	1	7.5
onset_LA	Fraction of T	0.8 ± 0.052	-0.011 (-0.065, 0.043)	0.91	2.9
onset_LV	Fraction of T	-0.062 ± 0.023	0.0075 (-0.037, 0.052)	0.43	29

Table 4: Repeatability of measured net flow volumes measured in mL in the mitral valve (MV), aortic valve (AV), and ascending aorta (AA) comparing intra-, inter- observer analysis and the SGRE and EPI sequences. For each location, the mean and standard deviation (SD), the mean difference and the limits of agreement ($\bar{d} \pm 1.96SD$), the p-value comparing the results from the two analyses/observers/sequences, and the coefficient of variation (CoV) are presented.

**Intra-observer variability**				
Location	*Descriptive Statistics Mean ± SD*	*Limits of agreement $\bar{d}$ (-1.96*SD, +1.96*SD)*	*P value*	*CoV (%)*
MV	84 +- 20	0.53 (-11, 12)	0.91	4.1
AV	88 +- 18	0.27 (-1.6, 3.2)	0.79	1.2
AA	85 +- 21	0 (-3.4, 4)	0.91	1.8
**Inter-observer variability**				
Location	*Descriptive Statistics Mean ± SD*	*Limits of agreement $\bar{d}$ (-1.96*SD, +1.96*SD)*	*P value*	*CoV (%)*
MV	82 +- 20	5.5 (-14, 25)	0.62	9.1
AV	89 +- 19	-1.2 (-8.9, 6.5)	0.85	2.7
AA	86 +- 21	-0.27 (-8.3, 7.7)	0.91	3.4
**Inter-sequence variability (SGRE-EPI)**				
Location	*Descriptive Statistics Mean ± SD*	*Limits of agreement $\bar{d}$ (-1.96*SD, +1.96*SD)*	*P value*	*CoV (%)*
MV	80 +- 22	-2.5 (-35, 30)	0.91	13
AV	87 +- 19	6.5 (-16, 29)	0.52	9.1
AA	86 +- 20	-0.088 (-10, 9.9)	0.97	4.2

Sensitivity analysis

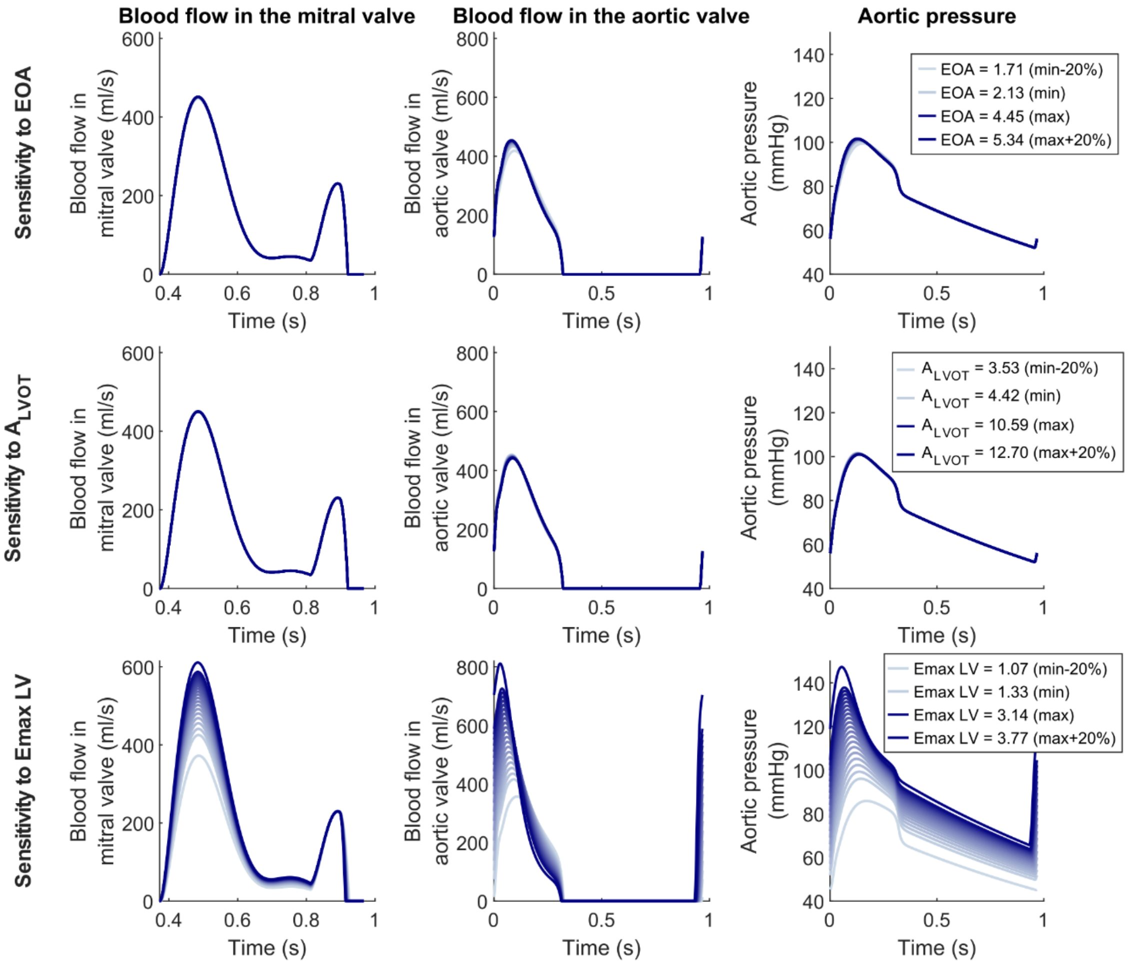

Figure 1: Sensitivity analysis of the effect of changes in the model parameters EOA (row 1), ALVOT (row 2), and Emax_LV (row 3) on the model simulations of blood flow in the mitral valve (column 1), blood flow in the aortic valve (column 2), and blood pressure in the aorta (column 3).

Method:

To investigate the robustness of the model to the input parameters, a sensitivity analysis was performed by changing the values of each of the input parameters separately and studying the effect on the model simulations. The investigated input parameters were the effective orifice area of the aortic valve (EOA), the left ventricular outflow tract area (A_{LVOT}), and the maximum elastance of the left ventricle (Emax LV). The maximum elastance is not directly an input parameter, but it is calculated from other measurements such as the left ventricular end systolic volume. For each parameter, several simulations were performed with the investigated parameter set to a range of values while all other parameter values were fixed to values estimated from data by observer 1 from one of the 10 healthy subjects. Each parameter was simulated with a range of 20 values between the minimum and maximum values found among all subjects and all observers and sequences, and with +20% of the maximum value and -20% of the minimum value, thus evaluating the effects of a large variability in the input parameters.

Result:

The resulting model simulations are shown in Figure 1. When changing the EOA (row 1), small effects on the blood flow in the aortic valve and aortic pressure are seen, while no effect is observed in the mitral valve. The effects of A_{LVOT} (row 2) are similar, with even smaller effects on the aortic simulations. The effects of changes in Emax_LV (row 3) are larger, with effects on the amplitude of the early peak in the mitral valve and the amplitudes of the flow and pressure in the aorta.
